# RNA-binding proteins to assess gene expression states of co-cultivated cells in response to tumor cells

**DOI:** 10.1186/1476-4598-3-24

**Published:** 2004-09-07

**Authors:** Luiz OF Penalva, Michael D Burdick, Simon M Lin, Hedwig Sutterluety, Jack D Keene

**Affiliations:** 1 Department of Molecular Genetics and Microbiology, Center for RNA biology, 414 Jones Building, Research Drive, Duke University Medical Center, Durham, North Carolina 27710 USA; 2 Duke Bioinformatics Shared Resource, Duke University Medical Center, Durham, North Carolina 27710 USA; 3 Children's Cancer Research Institute. UTHSCSA. Mail code 7784 7703 Floyd Curl Dr. San Antonio, TX 78229-3900 USA; 4 Bayer Corporation 85 T.W. Alexander Drive Research Triangle Park NC 27709 USA; 5 Institut fur Krebsforschung Abt. Fur Angewandte und Experimentelle Onkologie Borschkegasse 8a A-1090 Wien Austria

## Abstract

**Background:**

Tumors and complex tissues consist of mixtures of communicating cells that differ significantly in their gene expression status. In order to understand how different cell types influence one another's gene expression, it will be necessary to monitor the mRNA profiles of each cell type independently and to dissect the mechanisms that regulate their gene expression outcomes.

**Results:**

In order to approach these questions, we have used RNA-binding proteins such as ELAV/Hu, poly (A) binding protein (PABP) and cap-binding protein (eIF-4E) as reporters of gene expression. Here we demonstrate that the epitope-tagged RNA binding protein, PABP, expressed separately in tumor cells and endothelial cells can be used to discriminate their respective mRNA targets from mixtures of these cells without significant mRNA reassortment or exchange. Moreover, using this approach we identify a set of endothelial genes that respond to the presence of co-cultured breast tumor cells.

**Conclusion:**

RNA-binding proteins can be used as reporters to elucidate components of operational mRNA networks and operons involved in regulating cell-type specific gene expression in tissues and tumors.

## Background

Many recent studies have described the use of microarrays to identify genes expressed in different types of cancers (reviewed in [1,2]. Most of these transcriptomic studies monitor the steady state levels of expressed mRNAs in order to derive the "molecular signatures" of tumors [2]. However, the gene expression profile of a whole tumor corresponds to the combined profiles of the different cell types contained within it (e.g. endothelial cells, T-cells, cancer cells, stromal cells, etc.). Moreover, the multiple cell types present in a tumor or organ are interdependent and exchange biochemical signals as a means of cell-cell communication [3]. An important example of cell-cell communication is evident in angiogenesis, the mechanism by which new blood vessels vascularize tumors and other organs (reviewed in [4]). Monitoring the dynamics of gene expression in each cell type of a tumor during angiogenesis will advance understanding of tumorigenesis as well as organogenesis, in general.

Methods have been devised to generate mRNA samples from specific types of tumor cells. These include microdissection, laser capture (reviewed in [4-6], and cell sorting based on specific membrane markers [7]. Here we demonstrate that RNA-binding proteins can be used to isolate mRNA populations representing total cell mRNA from specific types of cells, as well as discrete mRNA subpopulations that represent post-transcriptionally regulated subsets of mRNAs that encode functionally related proteins. We propose that these represent genes whose regulation is important for tumor growth and maintenance.

RNA binding proteins play a key role in post-transcriptional regulation, participating in splicing, mRNA transport and localization, mRNA stability and translation (for overview see ref. [8]). Our lab has devised biochemical and immunological approaches to gene expression profiling by using RNA-binding proteins as reporters of discrete mRNA subsets in metazoan cells [8-10]. For example, we identified subpopulations of mRNAs that are associated with ELAV/Hu RNA-binding proteins that are expressed in specific cell types [10]. While we and other labs have demonstrated the isolation of mRNA subsets that are potentially co-regulated using RNA binding proteins as reporters of gene expression, methods have not been described that provide information about coordinated posttranscriptional regulation within specific types of cells during tumorigenesis and development. Moreover, because many different mRNA-binding proteins in specific cell types are known to interact with unique subpopulations of mRNAs encoding functionally related proteins [9-15] they can be informative of the dynamic effects of cells on one another. Therefore, it will be necessary to assess changes in gene expression that occur when cells such as tumor cells and endothelial cells interact in order to understand growth control and critical processes such as angiogenesis.

In this study, we define a model system for using poly (A) binding protein (PABP) to recover mRNAs from specific cell-types in mixed cell cultures. Using this approach, we were able to determine how the gene expression profiles of endothelial cells change in response to the presence of breast cancer cells. Among the advantages of this approach are: a) no manipulations or treatments are required prior to the preparation of cell extracts, b) the recovered mRNA population can be identified directly using genomic methods, and c) RNA binding proteins can be engineered for expression in different cell types using various molecular tags in order to discriminate cell-specific mRNA populations. These studies provide a methodological basis for creating mouse models in which different types of cells within a tumor express RNA binding proteins to reveal unique populations of posttranscriptionally regulated mRNAs.

## Results and Discussion

The goals of these experiments are to validate procedures for the isolation and characterization of discrete mRNA sub-populations associated with RNA binding proteins expressed in specific cell types within a tumor or organ in order to assess the responses of cells to their surroundings. Earlier studies have shown that mRNA subpopulations in single cell types reflect the functions of the RNA binding proteins with which they associate and can provide key information about post-transcriptional regulatory mechanisms of gene expression [8-11,13,15-18]. In model organisms, such information can be obtained by expressing epitope-tagged RNA binding proteins using tissue-specific promoters [19] or by using virus-specific receptors (M.D.B., L.O.F.P. and J.D.K. unpublished). In this study we demonstrate the feasibility of this approach by using two different cell types in culture that each express specific RNA binding proteins as reporters of gene expression profiles.

### Comparison of total mRNA of PY4.1 endothelial cells with their PABP-associated mRNA patterns

**Figure 1:**
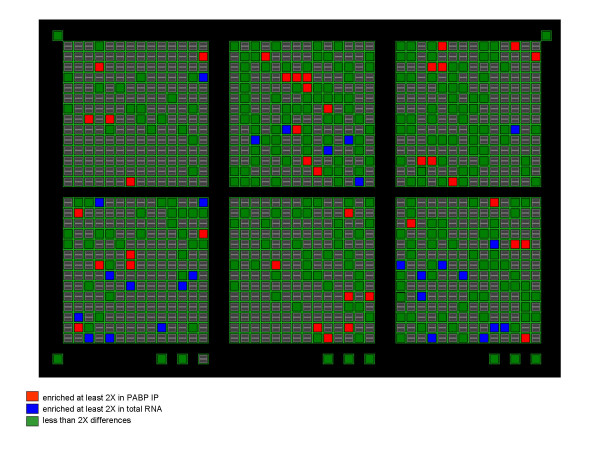
**Comparison of the gene expression profiles of total RNA and PABP-associated mRNA populations. **Total RNA from PY4.1 murine endothelial cells and mRNA immunoprecipitated from cell extracts using anti-PABP serum were radiolabeled and hybridized on 1.2 mouse Atlas arrays (CLONTECH). The image overlay comparing total RNA with PABP-associated gene expression profiles was derived using the Atlas software with a global normalization showing quantitative, but not qualitative differences.

Microarray analysis was used to compare the gene expression profiles obtained using total RNA and PABP-associated RNA of PY 4.1 endothelial cells (Figure 1 and supplementary data). These patterns were highly reproducible and consistent. Very few if any qualitative differences were observed when comparing these mRNA patterns indicating that the same set of expressed genes was detected in both preparations. However, quantitative differences were observed between some of the PABP-associated mRNA levels and those of the total mRNA population (Figure 1 and supplementary data). Approximately 19% of the expressed mRNAs varied more than two fold. In the case of the total mRNA profile (transcriptome), the signal intensity reflects the steady-state level of each mRNA, while the signal intensity of the PABP associated messages likely correlates with their translational activity [20]. PABP is an essential RNA binding protein that is highly conserved among eukaryotic organisms. It mediates interactions between polyadenylated mRNA sequences at the 3' ends of mRNAs and the eIF-4G protein [21]. Interactions of eIF-4G with the cap-binding protein, eIF-4E, are believed to circularize the mRNA and to prepare it for association with ribosomes. Many studies have shown that PABP is involved in activating the stability and translation of mRNAs to which it is bound (reviewed in [22,23], by protecting the poly (A) tail from exonuclease attack [24], preventing mRNA decapping [25], by promoting mRNA maturation [26] and by stimulating the initiation of translation [20]. Most studies are in general agreement that PABP functions as a translational activator by facilitating the assembly of mRNAs and ribosomes. On the average, a single PABP is expected to recognize approximately fifteen adenylate residues, suggesting that approximately ten molecules of PABP are bound to the average mRNA that has a poly (A) stretch of 150–200 in length [27].

Several reports indicate that substantial differences can be found when comparing the steady state levels of mRNAs (transcriptome) with proteins (proteome) in the same cell population [28,29]. The accumulated levels of some proteins and their corresponding mRNAs can vary by as much as 30-fold [28-30]. The differential between steady state levels of mRNA and protein are expected to be more dramatic under conditions in which post-transcriptional regulation plays a major role. For example, following T cell-activation or during neuronal differentiation, translational control is thought to affect a significant proportion of the proteomic outcome [31,32].

It is possible that gene expression profiles obtained by immunoprecipitating mRNA-PABP complexes may reflect the functional state of protein production from these mRNAs [10]. For the purposes of this study, PABP is used as a functionally relevant RNA-binding protein with which to compare changes in bound mRNAs across gene expression profiles.

### Expression of tagged PABP does not interfere with cell growth

**Figure 2:**
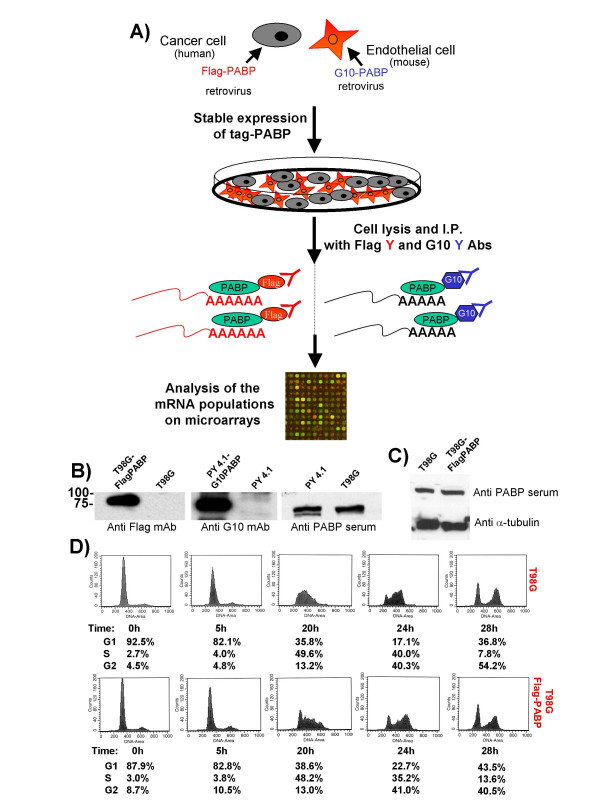
**A) Experimental design for sorting cell type specific mRNA populations using RNA binding proteins in mixed cell cultures**. Two cell lines from two different species (murine endothelial PY4.1 and human glioma tumor T98G) are engineered to express G10-tagged and FLAG-tagged PABP, respectively. Cell-type specific gene expression profiles are obtained from co-cultured cells or mixed cell extracts after immunoprecipitation with specific antibodies against the different tags. The RNA samples are phenol extracted, precipitated and subsequently analyzed by RPAs or microarrays. **B) Western blots of cell lines expressing tagged-PABP. **Immunoblots of extracts from T98G cells expressing Flag-PABP were probed with anti-Flag antibody, while extracts from PY4.1 and PY4.1 cells expressing G10-PABP were probed with anti-G10 antibody. Control blots of extracts of T98G and PY4.1 cells with anti-sera against PABP. **C) Comparison between the overall levels of PABP of T98G cells and T98G cells expressing Flag-PABP**. Immunoblots of extracts from T98G cells and T98G cells expressing Flag-PABP were probed with anti-sera against PABP and anti-α tubulin antibody, as a loading control. **D) Comparison of the cell cycle status of T98G and T98G expressing Flag-PABP cells **– Cells were arrested at G0/G1 by serum deprivation and stimulated to re-enter the cell cycle by addition of serum. At the indicated times, aliquots of cells were processed for FACS analysis to determine the population distribution in G1, S, and G2 stages of the cell cycle. No differences between of the cell cycle of T98G cells and T98G cells expressing Flag-PABP were observed.

In order to compare mRNA profiles from mixed cell populations, we prepared two different cell lines stably expressing different epitope tagged PABPs. Figure 2 outlines the experimental approach. T98G human glioma cells and PY4.1 mouse endothelial cells were co-cultured, cell extracts were prepared and antibodies against PABP, Flag-PABP and G10-PABP were used to immunoprecipitate the mRNP complexes in order to generate mRNA populations for gene expression analysis. Concerns that the epitopes represented in the tags might affect the results were addressed using PY4.1 cells stably expressing either Flag-tagged PABP or G10-tagged PABP. Cell extracts from both of the stable cell lines grown separately were prepared and immunoprecipitated with the respective antibodies. The two mRNA populations generated by this procedure were compared using an RNAse protection assay (RPA) and a microarray analysis. The results showed no significant qualitative or quantitative differences between these profiles in that 96% of the genes detected by microarray were within 1.5-fold of one another (data not shown).

A potential complication for this type of analysis is that expression of a tagged-RNA binding protein, in this case PABP, could affect cell growth. While these cell lines appeared unaffected morphologically, the levels of PABP in cell lines expressing tagged-PABP and respective control cell lines were evaluated and compared by Western blotting. No substantial change in overall PABP expression was observed when expressing exogenous tagged PABP (Figure 2C). This result was expected, since PABP has been shown to inhibit the translation of its own mRNA by binding to poly (A) sequences found in the 5' UTR. This fortuitous auto-regulatory mechanism is believed to keep the level of PABP constant in the cell, thereby avoiding excessive overexpression [33].

No changes in cell growth or mortality of the cell lines used in this study or in other cells lines expressing tagged-PABP were observed. Moreover, the cell cycle kinetics of T98G cells expressing Flag-PABP and cells that were subsequently stimulated by serum addition were compared to those of T98G cells containing the empty vector, pCMVneo and no substantial differences were observed using fluorescent cell sorting (Figure 2D). We conclude that expression of neither the authentic PABP, nor the tagged-PABP has untoward effects on the growth and homeostasis of these cells.

### PABP does not exchange between mRNAs in cell extracts

**Figure 3:**
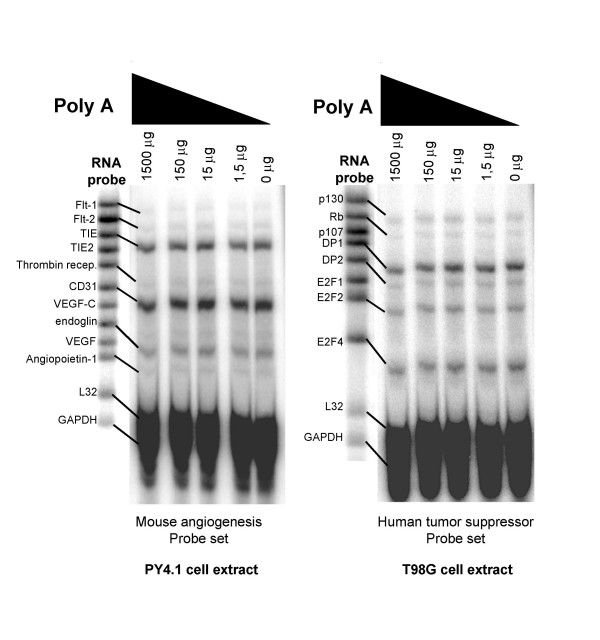
**Reassortment of mRNA and PABP was not detected in cell lysates. **The potential for displacement of PABP was tested by adding increasing amounts (0–1500 μg) of pure competitor poly (A) to 400 μl of cell lysates of PY4.1 and T98G cells prior to incubation with antibody-coated (anti-PABP) beads. Following immunoprecipitation, mRNAs were isolated from the pellets and analyzed using the RNase Protection Assay (RPA) mouse angiogenesis (mAngio) and human tumor suppressor (hTS1) multi-probe sets. Unprotected RNA probes were used to identify the nature of the different sized protected fragments. The experiment shows that the interaction between PABP and the endogenous mRNA targets cannot be disrupted by competing poly (A) RNA.

Early studies of PABP binding to mRNA indicated that a dynamic exchange or hopping of PABP from mRNA to mRNA might be an important aspect of its function [27,34]. For the expression profiling methods described above to be precise, it is critical to avoid post-lysis exchange (or adventitious reassortment) of PABP with mRNAs. In other words, does free mRNA in a cell extract displace the mRNA originally bound to PABP during the incubation period; or instead, does free PABP in an extract exchange by binding to available mRNAs? PABP is a good test model in this case because it has been suggested to "hop" based on in vitro studies, and it is a highly abundant RNA-binding protein. To examine these possibilities, we added increasing amounts of competitor poly (A) RNA with an average length of 550 nucleotides to lysates of mouse endothelial PY4.1 and human glioma T98G cells. After immunoprecipitation with anti-PABP serum, mRNAs were isolated from the pellets and analyzed using a highly sensitive multi-probe RNase Protection Assay (RPA) as described previously [10]. Figure 3 shows that the mRNAs originally bound to PABP were not displaced by the competing poly (A) RNA even at concentrations as high as 1000 fold excess. The inability of free poly (A) RNA to compete bound PABP off of endogenous mRNA reflects a stable interaction between PABP and endogenous mRNA. These data suggest that exchange of mRNA into PABP RNPs is not likely to distort gene expression profiles obtained by immunoprecipitating PABP, and in addition, this observation is not compatible with a previous "hopping model" for PABP [27].

We have also addressed the potential problem of having a pool of free PABP in an extract that could be available to bind mRNA during incubation. Sucrose gradient analysis indicated that this is very unlikely since the majority of PABP was found in heavy polysomes and associated with mRNA, while only a small percentage was found in the upper portion of the sucrose gradients (H.S. and J.D.K., unpublished data). In total, these results demonstrate that reassortment of PABP in these cell extracts was not a significant limitation to using PABP RNPs for gene expression profiling of bound mRNAs.

As noted above, it has been suggested that yeast PABP uses a "hopping" mechanism in vivo by moving from RNA to RNA [27,34]. While this experiment is not a direct test of that hypothesis, these data are not consistent with a hopping or exchange of PABP among the mRNAs in our cell extracts, but suggest instead that PABP forms a stable RNP complex with polyadenylated transcripts.

### Detection of cell-specific mRNAs using epitope-tagged PABP

**Figure 4:**
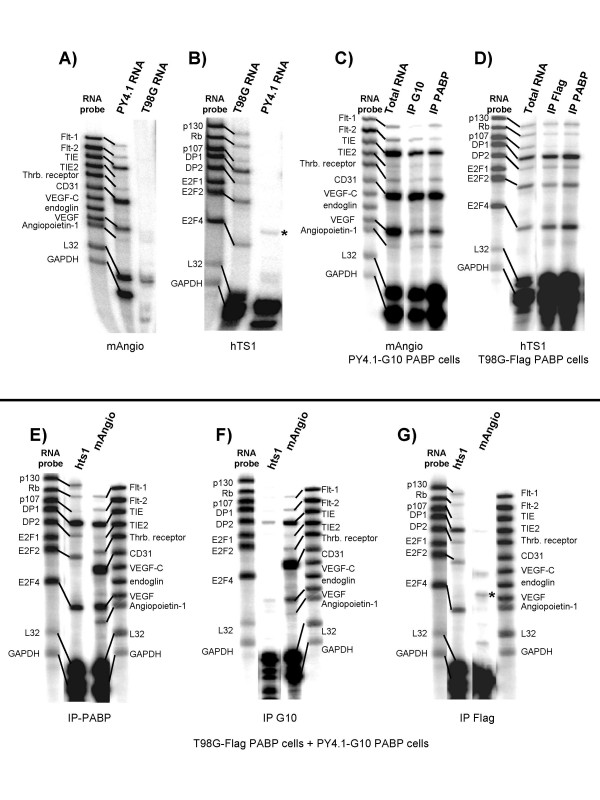
**RNase Protection Assay (RPA) of mRNAs from mixed mouse and human cell lines**. Species specificity of multiprobe RPAs using: **A) **the human tumor suppressor probe set (hTS1), and **B) **the mouse angiogenesis RPA probe set (mAngio), was verified using total RNA extracted from murine PY4.1 cells and human T98G cells. Unprotected RNA probes were used to identify the nature of the different sized protected fragments. **C) **RPA gene expression profile of PY4.1 cells expressing G10-PABP obtained with total RNA and with mRNA derived from immunoprecipitations with anti-PABP serum or anti-G10 antibody **D) **RPA gene expression profile of T98G cells expressing Flag-PABP obtained from total RNA and from mRNA derived from immunoprecipitations with anti-PABP serum or anti-Flag antibody. **E, F and G) **Mixed extracts from cells expressing T98G Flag-PABP and PY4.1 G10-PABP were immunoprecipitated with anti-PABP serum or anti-Flag or anti-G10 antibodies. The mRNA populations generated by immunoprecipitations were analyzed with RPA of both the mouse angiogenesis (mAngio) and the human tumor suppressor probe (hTS1) sets. GAPDH and L32 are controls in both probe sets and show cross species hybridization. The asterisk in B and G also indicate a band resulting from cross species hybridization. The experiments indicate that species-specific mRNA populations can be isolated and quantified by the use of distinct tagged-PABPs.

The question of whether reassortment of PABP occurs among mRNAs in cell extracts was also examined using lysates from mixed mouse and human cells. We used two different cell lines, murine endothelial PY4.1 and human glioblastoma T98G, that express G10-PABP and Flag-PABP, respectively (Figure 2B). T98G cells have a volume approximately 3 to 4 times larger than PY4.1 cells. These cell lines were co-cultured in an approximate 1:1 cell ratio and subsequently lysed, or in separate experiments, lysates of each were mixed at equivalent amounts of total protein prior to immunoprecipitation. Both approaches gave the same results. Using the G10 antibody to precipitate only PY4.1 mRNAs and Flag antibody to precipitate T98G mRNAs we were able to examine the separate populations using a multiprobe RPA (Figure 4). Thus, by mixing a human (T98G) and a murine (PY4.1) cell line we could take advantage of the species-specific RPA probe sets. Given our interest in tumor angiogenesis, we used mouse angiogenesis (mAngio) and human tumor suppressor (hTS1) RPA probe sets after they were tested for cross species hybridization. Both probe sets showed good specificity of discrimination with the exception of the L32 and GAPDH control genes as expected (Figure 4A and 4B). In Figure 4C and 4D, it is apparent that the same expressed genes were detected whether using total RNA, or mRNA obtained by immunoprecipitation with anti-PABP, anti-Flag or anti-G10 antibodies. Extracts from mixed cell lines were immunoprecipitated and analyzed using both human and mouse probe sets (Figure 4E,4F and 4G). Both mouse and human mRNAs were detected when anti-PABP rabbit serum was used to precipitate both endogenous and exogenous PABP, while immunoprecipitations with anti-G10 and anti-Flag antibodies enriched the mRNA population for each species with only minor background from the other species. While the discrimination obtained in these experiments was excellent, a low degree of background due to non-specific binding of mRNA to the agarose beads was consistently observed even in the absence of antibody.

### Analysis of PABP-associated mRNA populations using microarrays

**Figure 5:**
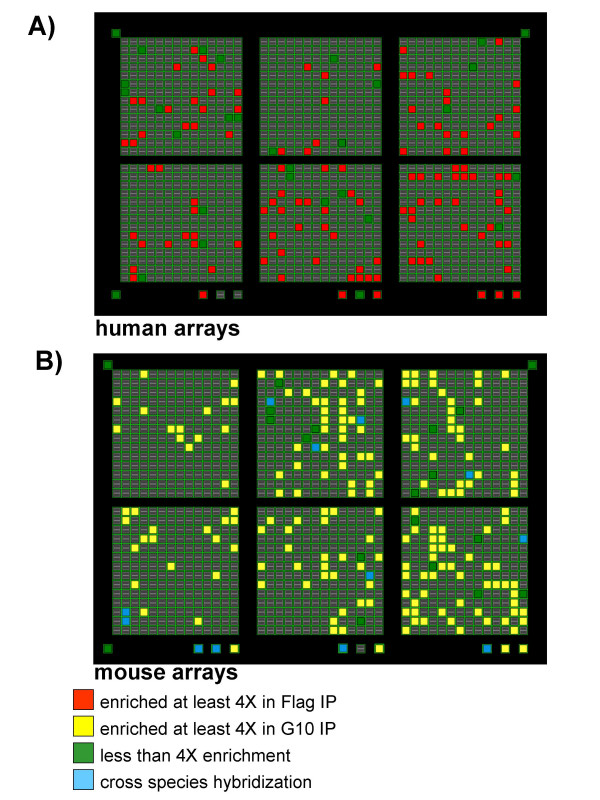
**Discrimination of the gene expression profiles of mixed human and mouse cell lines using microarrays. **Cell extracts from T98G Flag-PABP and PY4.1 G10-PABP cells were prepared, mixed and immunoprecipitated with both anti-Flag and anti-G10 antibodies. The mRNA populations generated by both immunoprecipitations were analyzed on human and mouse 1.2 CLONTECH arrays. When anti-Flag antibodies were used, the T98G mRNA population was enriched in relation to the PY4.1 mRNA population. When anti-G10 antibodies were used, the PY4.1 mRNA population was enriched in relation to the T98G mRNA population.

In order to evaluate the degree of mRNA enrichment over background using a genome–wide methodology, we analyzed immunoprecipitated mRNAs from mixed cell populations on CLONTECH Atlas arrays. We first tested these arrays for cross species hybridization using total RNA and it was minor (data not shown). The mouse RNA on human Atlas arrays did not show any detectable cross hybridization signal, while the human RNA on mouse Atlas arrays showed a small percent (2–3%) of cross hybridizing mRNAs (Figure 5B, blue squares).

To identify the PABP-associated mRNAs in the T98G Flag-PABP and PY4.1 G10-PABP cells, extracts were prepared as described above, followed by immunoprecipitation with either anti-Flag or anti-G10 antibodies. The mRNA populations generated by both immunoprecipitations were analyzed on human and mouse 1.2 Atlas arrays. Cross-species hybridization was monitored and genes showing cross-species reactivity were eliminated from consideration. A comparison of Flag versus G10 PABP-associated mRNAs was performed to assess the degree of enrichment. In an average experiment for the mouse genes, 91 % (184 out of 202 detected genes) were enriched at least 4 fold in the G10 PABP population when compared to the Flag PABP population. For the human genes, 82.4 % (122 out if 148) were enriched at least 4 fold in the Flag PABP population in relation to the G10 PABP population (Figure 5 and supplementary data).

### Changes in gene expression induced by co-cultivation of PY4.1 endothelial cells with 4T1 breast cancer cells

Having demonstrated that the approach we described using PABP can be used to efficiently recover cell type specific mRNAs from mixed cell types, we addressed the consequences of cell-cell communication and changes in gene expression that were induced in the endothelial cells by co-cultivation with the tumor cell. The goal of these experiments is to gain insight into how endothelial cells respond to the presence of cancer cells in cell culture as a first approximation of changes in gene expression that may be involved in early stages of angiogenesis. We used two murine cell lines, the PY4.1 line described above and a 4T1 breast tumor cell line that can produce tumors and spread by metastasis in nude mice.

**Figure 6:**
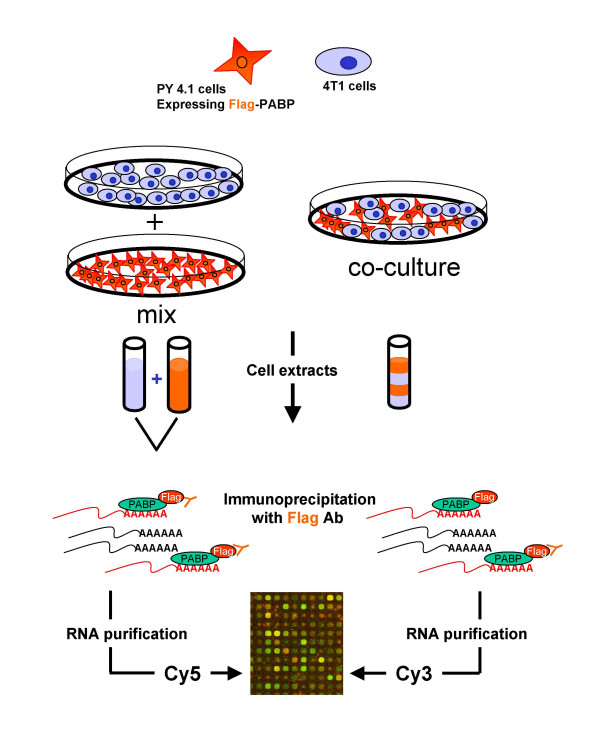
**Effects of co-cultivated mouse tumor cells on gene expression profile of mouse endothelial cells. **PY4.1 cells expressing Flag-PABP and 4T1 cells were grown either separately or together in a co-culture. Cell extracts from the co-culture and from a mixture of the monocultures were prepared. Immunoprecipitation of extracts with anti-Flag antibodies generated two distinct PY4.1 cells mRNA populations that were compared by microarray. The comparison revealed PY4.1 cellular genes that changed their gene expression profile in response to presence of 4T1 breast cancer cells.

Figure 6 represents a schematic view of two branches of our experimental strategy. In the first line of inquiry, PY4.1 cells stably expressing Flag-PABP and 4T1 cells were co-cultured. The number of plated cells of each type was calculated based upon measurements of their different growth rates. After 48 hours the co-cultured cells reached confluence. Approximately 50% of the petri dish surface was covered with PY4.1 cells and 50% was covered with 4T1 cells. Cells were harvested and extracts were prepared as described above. In the second line of inquiry, the two cell types were plated separately. Cells were harvested after 48 hours of incubation, the extracts were prepared, and mixed proportionally to those used in the first experimental line of investigation. Subsequently, extracts from the co-cultured and the mixed cells were immunoprecipitated with anti-Flag antibodies and mRNAs analyzed on microarrays. The comparison between the two mRNA populations (co-culture versus mix) was used to detect changes in the gene expression profile of PY4.1 cells as a response to the presence of 4T1 cells. In a separate experiment, a comparison between PY4.1 total RNA labeled with Cy3 and Cy5 was performed to rule out dye bias (not shown).

**Figure 7:**
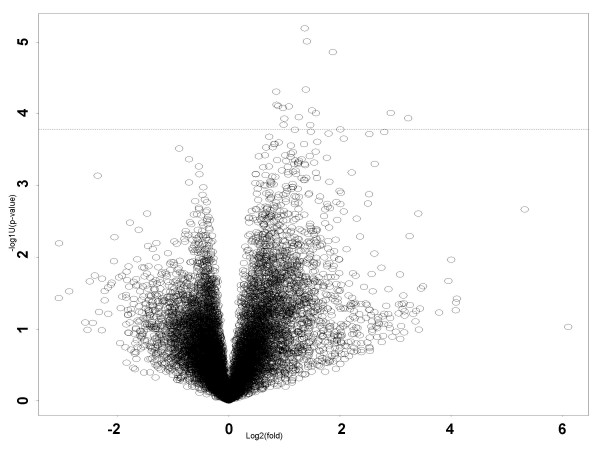
**"Volcano" plot of p-value versus fold change in expression level. **Dashed line indicates the cutoff of the top 20 enriched genes shown in Table 1.

As expected, the great majority of the genes expressed in PY4.1 cells were not altered in their levels of expression while in co-culture. Of interest, a small number of genes were consistently upregulated in four independent experiments. To assess the consistency of the fold change, we plotted the p-value (from modified t-test) against the average fold change (Figure 7). A 'volcano plot' summarizes both the magnitude of change and the corresponding statistical significance for all genes. We sorted the candidate genes according to their p-values (Table 1), and the top-20 genes were identified and are listed along with comments concerning their biological function(s). [See Supplementary Data for the complete microarray analysis – ]. Several of the genes present in this population are gene expression regulators that fall into two major categories: RNA binding proteins and DNA binding proteins/transcription factors. We expected to find gene expression regulators as part of an early response to cell surface interactions or secreted factors from the other cell line. As with any biochemical cascade event, changes in the expression of global regulators as well as structural genes (such as those encoding membrane or cytoskeletal proteins) often precede downstream alterations in the expression of other important genes. Among the RNA binding proteins identified in our screen were stem-loop binding protein, a highly conserved RNA binding protein that binds a stem loop structure in the 3'UTR of histone mRNAs and is required for both processing and translation of histone messages [35], Brul4, the mouse homologue of *Drosophila bruno*, a translation repressor which functions at the early steps of embryogenesis [36], and quaking, described as a key gene involved in the myelination of the central nervous system and other regulatory functions [37]. It should be noted that we did not identify PY4.1 genes whose expression decreased as a response to the presence of 4T1 cells.

**Table 1:** List of the top 20 PY4.1 genes that were upregulated in response to the presence of 4T1 tumor cells. Genes are classified according to their biological function. Gene expression regulators (GR). Genes involved in metabolism (M). Genes related to cell cycle or cell division (C). Genes encoding structural proteins (S). Other genes (O).

**Name, classification**	**gbID**	**fold**	**P value**	**biological function**
**1**-Heterogeneous nuclear ribonucleoproteinH1, **(G R)**	NM_021510	2.6	6.40E-06	RNA binding, RNA processing and modification
**2**-High mobility group box 1, **(G R)**	NM_010439	2.7	9.78E-06	DNA binding, nitric oxide biosynthesis, inflammation mediator, cell differentiation
**3**-Prothymosinalpha, **(C)**	NM_008972	3.7	1.38E-05	cell proliferation, cell division
**4**-RIKEN cDNA2610016F04 gene, **(G R)**	AK009120	2.6	4.59E-05	putative DNA binding, transcritionfactor
**5**-ATPase, H+ transporting, **(M)**	NM_024173	1.8	4.92E-05	hydrogen-exporting, ATPaseactivity, phosphorylativemechanism
**6**-RIKEN cDNA2510010F10 gene, **(O)**	AF215660	1.8	7.45E-05	described as a carnitinedeficiency-associated gene
**7**-Stem-loop binding protein, **(G R)**	NM_009193	2.1	7.88E-05	RNA binding, histonemRNA processing
**8**-Quaking, **(G R)**	NM_021881	1.9	7.79E-05	RNA binding, participates in myelination
**9**-RIKEN cDNA2410004I17 gene, **(O)**	AK010391	2	8.26E-05	unknown
**10**-Purinerich element binding protein A, **(G R**)	NM_008989	2.8	8.95E-05	DNA and RNA binding, association with rough endoplasmic reticulum, postnatal brain development
**11**-Similar to isopentenyl-diphosphatedelta isomerase, **(M)**	BC004801	3	9.80E-05	cholesterol biosynthesis, steroid biosynthesis
**12**-P53 apoptosis effectorrelated to Pmp22, **(O)**	NM_022032	2.4	1.11E-04	induction of apoptosis
**13**-Tumor differentially expressed 1, like, **(S)**	NM_019760	2	1.17E-04	plasma membrane
**14**-RIKEN cDNA5830409B12 gene, **(S)**	AK017914	7.6	9.73E-05	putative cytoskeleton associated protein
**15**-G7e protein, **(S)**	NM_033075	2	1.42E-04	resembles viral envelope genes
**16**-Procollagenlysine, 2-oxoglutarate 5-dioxygenase 2, **(M)**	NM_011961	2.8	1.43E-04	protein metabolism
**17**-Receptor-like tyrosine kinase, **(M)**	L21707	1.7	2.08E-04	ATP binding, kinaseactivity
**18**-RIKEN cDNA4930506D01 gene, **(G R)**	BC006745	2.8	1.77E-04	putative transcription factor
**19**-MusmusculusBRUL4 (Brul4) mRNA, **(G R)**	AF314173	5.8	1.91E-04	RNA binding, translation regulator
**20**-CyclinI, **(C)**	NM_017367	2.3	1.67E-04	cell cycle, cyclin-dependent protein kinaseregulator activity
**relevant references**	
**1**-none				
**2**-Gastroenterology. 2002. 123:790–802; J LeukocBiol. 2002. 72:1084–1091				
**3**-Peptides. 2000. 21:1433–1446; IntJ Biochem Cell Biol. 1999. 31:1243–1248.				
**4**-none				
**5**-J BiolChem. 2002. 277:36296–36303; Gene. 2003. 302: 147–153.				
**6**-Biochim BiophysActa. 2002. 1577:437–444.				
**7**-J. Cell Sci. 2002. 115: 4577–4586.				
**8**-Neuron. 2002. 36:815–829; Nucleic Acids Res. 2003.31:4616–4624				
**9**-none				
**10**-J BiolChem. 2002.277:37804–37810; Mol Cell Biol. 2003. 23:6857–6875				
**11**-J Mol Evol. 2003. 57: 282–291.				
**12**-Genes Dev. 2000. 14: 704–718.				
**13**-J Exp Biol. 2000. 203:447–457.				
**14**-none				
**15**-Genomics. 1996. 15:5–12				
**16**-J BiolChem. 2003. 278:40967–40972.				
**17**-none				
**18**-J Immunol. 1995. 154:1157–1166; Proc NatlAcadSciU S A. 1992. 89:11818–11822.				
**19**-CytogenetGenome Res. 2002.97:254–260.				
**20**-Gene. 2000. 256: 59–67.				

The presence of several RNA binding proteins among the top-20 genes affected by co-cultivation may result in downstream effects on gene expression, and we plan to examine the target mRNAs of these RNA binding proteins in endothelial cells. This should help elucidate additional post-transcriptional pathways and networks regulating cell growth mechanisms and tumorigenesis [9].

## Conclusion

This study describes changes in the gene expression profile of an endothelial cell when co-cultivated with a tumor cell by isolating ribonucleoprotein complexes and identifying their associated mRNAs using genomic arrays. Moreover, it presents a model system that can be used to elucidate post-transcriptional operons in specific types of cells by using various RNA binding proteins from mixed cell cultures as a novel approach to understanding how cell-cell communication affects gene expression during tumorigenesis and organogenesis.

## Methods

### Cell lines and media

Murine endothelial PY4.1 cells were kindly provided by Dr. Christopher Kontos, Duke University Medical Center. Human gliobastoma T98G and murine breast cancer 4T1 cells were obtained from American Type Culture Collection. All cell lines were maintained in DMEM Medium (Gibco) supplemented with 10% Fetal Bovine serum.

### Constructs and stable cell lines

The ORF of human PABP I containing the Flag tag (GACTACAAGGACGACGATGACAAG) or the G10 tag (CCACCATGGCT AGCATGACTGGTGGACAGCAAATGGGT) at the 5' end was cloned into the pCMV-Neo retroviral vector [38]. Stable lines expressing the Flag-PABP (T98G and PY 4.1 cells) and the G10-PABP (PY 4.1 cells) were obtained according the protocol described in the Pantropic Retroviral Expression System (Clontech).

### Antibodies

Monoclonal anti-G10 antibodies were obtained as previously described [39]. Antibodies against Flag and α-tubulin were obtained from SIGMA. A PABP carboxy-terminal (last 172 amino acids) was prepared by cloning a PCR product into the pGEXCS expression vector. The protein was purified by their affinity to glutathione beads (Amersham Biosciences). The purified proteins were dialyzed against 1 × PBS, 20% glycerol and sent to COVANCE Inc., where a rabbit was immunized.

### Protein preparation and Western analysis

Protein extracts were prepared from T98G and PY4.1 cell lines by homogenization in polysomal lysis buffer [10]. 50 μg of extract were fractionated by electrophoresis in 10% polyacrylamide-SDS Laemmli gels. Proteins were transferred to nitrocellulose membranes using a transfer cell (Bio-Rad). After blocking with 5% nonfat milk in PBS-Tween 20 buffer, the membranes were incubated with anti-PABP rabbit serum (1:10,000 dilution), anti-Flag antibody (1:1,500 dilution) or anti-G10 antibody (1:10,000 dilution). Anti-rabbit or anti-mouse HPC IgGs (Amersham Biosciences) were used as secondary antibodies at a 1:3000 dilution. Blots were developed using an ECL detection kit (Amersham Biosciences) and exposed to film.

### Cell cycle experiments

Analysis of the cell cycle of T98G cells was performed as described [40].

### Immunoprecipitation of mRNP complexes from cell lysates

Cell lysates and immunoprecipitation of mRNP complexes were essentially performed as described [41]. Polyadenylated RNA (free poly-A) used in competition experiments was obtained from Amersham Biosciences.

### RNase Protection Assay

Total or immunoprecipitated RNAs were assayed by RNase protection by using the PharMingen Riboquant assay according to the manufacturer's recommendations (45014K). mAngio-1 (mouse angiogenesis) and hTS1 (human tumor suppressor) template sets were used (551418 and 556161, respectively). Protected riboprobe fragments were visualized on a phosphorimaging screen (Molecular Dynamics). Phosphorimages were scanned by using the Molecular Dynamics STORM 860SYSTEM at 100 μm resolution and analyzed by using Molecular Dynamics IMAGE QUANT software (version 5.0).

### Clontech microarrays, probing and analysis

cDNA array analysis was performed by using Atlas Mouse and Human 1.2 Arrays (CLONTECH). Probing of cDNA arrays was performed as described in the CLONTECH Atlas cDNA Expression Arrays User Manual (PT3140-1). Reverse-transcribed probes were radiolabeled with ^32^P α-dATP (Amersham Biosciences). After hybridization, the array membrane was washed and the results were visualized on a phosphorimaging screen (Molecular Dynamics). Phosphorimages were scanned by using the Molecular Dynamics STORM 860SYSTEM at 100 μm resolution and stored as .gel files. Images were analyzed by using ATLASIMAGE 2.01 software (CLONTECH). Global normalization was used when arrays being compared had approximately the same number of positive hits.

### Printed oligo arrays, probing and analysis

Printed oligo arrays using the Operon Mouse Oligo set version 2.0 (16,423 genes) were produced by the Duke Microarray Core Facility. Protocols used for preparation of slides, labeling, amplification, hybridization and scanning are described in .

GenePix data were normalized with pin-tip specific lowess normalization [42]. Differentially expressed genes were identified with a moderated t-test, which shrinks the estimated sample variances towards a pooled estimate [43]. This moderated t-test is more robust when the number of arrays is small. The candidate gene list is sorted by the p-value. All calculations were conducted using the bioconductor package [44].

## List of abreviations

ELAV – embryonic lethal abnormal vision

RPA – RNase Protection Assay

PABP – Poly A binding protein

## Author's contribution

LOFP was responsible for the experimental design and performed Western blots, immunoprecipitations, RPAs and microarray experiments. MDB helped perform RPAs and microarray experiments. SML performed statistic analysis of microarray data and prepared the webpage with supplementary data. HS generated the stable cell lines used in this study and performed the cell cycle experiment. JDK conceived the project and assisted in experimental design.

## Supplementary material


